# Diagnostic value of retrospective PET-MRI fusion in head-and-neck cancer

**DOI:** 10.1186/1471-2407-14-846

**Published:** 2014-11-19

**Authors:** Denys J Loeffelbein, Michael Souvatzoglou, Veronika Wankerl, Julia Dinges, Lucas M Ritschl, Thomas Mücke, Anja Pickhard, Matthias Eiber, Markus Schwaiger, Ambros J Beer

**Affiliations:** Department of Oral and Maxillofacial Surgery, Technische Universität München, Ismaningerstr. 22, D-82675 München, Germany; Department of Nuclear Medicine, Technische Universität München, Munich, Germany; Department of Otolaryngology, Head and Neck Surgery, Technische Universität München, Munich, Germany

**Keywords:** Multimodal imaging, PET-MRI fusion, Retrospective image fusion, Side-by-side analysis, Head-and-neck cancer, Staging

## Abstract

**Background:**

To assess the diagnostic value of retrospective PET-MRI fusion and to compare the results with side-by-side analysis and single modality use of PET and of MRI alone for locoregional tumour and nodal staging of head-and-neck cancer.

**Methods:**

Thirty-three patients with head-and-neck cancer underwent preoperative contrast-enhanced MRI and PET/CT for staging. The diagnostic data of MRI, PET, side-by-side analysis of MRI and PET images and retrospective PET-MRI fusion were systematically analysed for tumour and lymph node staging using receiver operating characteristic (ROC) analysis. The results were correlated to the histopathological evaluation.

**Results:**

The overall sensitivity/specificity for tumour staging for MRI, PET, side-by-side analysis and retrospective PET-MRI fusion was 79%/66%, 82%/100%, 86%/100% and 89%/100%, respectively. The overall sensitivity/specificity for nodal staging on a patient basis for MRI, PET, side-by-side analysis and PET-MRI fusion was 94%/64%, 94%/91%, 94%/82% and 94%/82%, respectively. MRI, PET, side-by-side analysis and retrospective image fusion were associated with correct diagnosis/over-staging/under-staging of N-staging in 70.4%/18.5%/11.1%, 81.5%/7.4%/11.1%, 81.5%/11.1%/7.4% and 81.5%/11.1%/7.4%, respectively.

ROC analysis showed no significant differences in tumor detection between the investigated methods. The Area Under the Curve (AUC) for MRI, PET, side-by-side analysis and retrospective PET-MRI fusion were 0.667/0.667/0.702/0.708 (p > 0.05). The most reliable technique in detection of cervical lymph node metastases was PET imaging (AUC: 0.95), followed by side-by-side analysis and retrospective image fusion technique (AUC: 0.941), which however, was not significantly better then the MRI (AUC 0.935; p > 0.05).

**Conclusions:**

We found a beneficial use of multimodal imaging, compared with MRI or PET imaging alone, particular in individual cases of recurrent tumour disease. Side-by-side analysis and retrospective image fusion analysis did not perform significantly differently.

## Background

Clinical examination and imaging is routinely performed for the staging of head-and-neck cancer (HNC) in order to establish the tumour extent and size, to assess nodal involvement, to evaluate detailed tumour spread, e.g. bone infiltration and perineural infiltration, and to identify distant metastasis [1]. Accurate imaging is essential for staging, treatment planning and follow-up, since surgical intervention and neo- or adjuvant therapy modalities depend on the outcome of the diagnostic results [2]. For head-and-neck tumours, magnetic resonance imaging (MRI) and computed tomography (CT) provide accurate anatomical information with good resolution but have well-known limitations, especially in the staging of the nodal involvement of the neck [3, 4] and recurrent disease [5, 6]. Furthermore, the differentiation between malignant bone infiltration and infectious bone reaction is also of poor accuracy [7]. An advantage of MRI is that fewer artefacts occur from (dental) metallic implants, which often interfere with CT interpretation [8].

Nowadays multimodal imaging by using positron emission tomography/computed tomography (PET/CT) has gained a wide acceptance as a powerful imaging tool, especially in recurrent tumour disease. The combination of morphological and functional imaging (multimodal imaging) has been shown to reduce false positive or false negative results [9, 10]. Driven by the success of PET/CT in HNC treatment [11, 12], hybrid PET/MRI scanners are now available to combine the high soft-tissue contrast of MRI with the molecular and/or metabolic information of PET. Previous reports have described the development of reliable PET/MR imaging protocols [10, 13–15] for HNC staging. However, to date, only a few sequential or fully integrated PET/MRI scanners are in clinical use. Most departments can still only offer the use of the two modalities (PET/CT and MRI) each as a single device. The separately gained images can be additionally analysed in two different ways: 1) side-by-side analysis and 2) retrospective software-based image fusion. The latter can be performed manually by shifting and rotating the images by using landmarks as reference [16, 17] or fully automatically [18]. Retrospective image fusion is, because of its complexity, time-consuming, hardware- and software-dependent and thus problematic in clinical routine. Furthermore, differences in the patient’s position during each imaging process limit the accuracy of the retrospective fusion. On the other hand, this method has been shown to lead to a more precise evaluation and treatment of HNC in complex cases [14, 19].

The purpose of this retrospective study was to assess the clinical value of retrospective PET-MRI fusion and to compare the diagnostic accuracy with side-by-side analysis MRI and ^18^F-FDG PET alone.

## Methods

### Ethics statement

The study has been performed in accordance with the Declaration of Helsinki and has been approved from an ethical and legal point of view by the ethical committee of the medical faculty of the Technische Universität München (registration number: 366/14).

### Patient population

Thirty-three patients (21 men and 12 women; age range 27–72 years; mean age 57 years) with primary malignant neoplasm (n = 23), recurrent tumour disease (n = 6) and lymph node metastasis in the framework of CUP (cancer of unknown primary) (n = 4) in the head-and-neck region were included in the study. All patients underwent clinically indicated preoperative contrast-enhanced MR imaging and ^18^F-FDG PET/CT imaging within 14 days (mean three days).

The suspicious lesions/tumours were localised in the oropharynx (n = 5), tongue (n = 5), floor of the mouth (n = 4), hypopharnyx (n = 4), buccal mucosa (n = 2), valleculla (n = 1), tonsil (n = 1) and salivary gland (n = 1). The recurrent tumours were localised in the floor of the mouth (n = 2), tongue (n = 1), parotid gland (n = 1), tonsil (n = 1) and oropharynx (n = 1) (Table 1).

**Table 1:** **Demographic data of the thirty-three enrolled patients, information concerning tumour location and study-relevant information as an overview**

Case	Location of tumour site	Time between MRT and PET (d)	Indication	Histopathological result	TNM classification
1	Tonsil	7	Primary staging	SCC	pT2 pN2b pMx
2	CUP/Tonsil	7	Primary staging	SCC	pT1 pN1 pMx
3	Oropharynx	1	Recurrent tumour	Chronic inflammation	-
4	CUP	3	Primary staging	SCC	cTx pN2b pMx
5	Tongue	0	Primary staging	SCC	pT2 pN2c pMx
6	Parotid Gland	8	Primary staging	SCC	pT3 pN2c pMx
7	Oropharynx	14	Primary staging	SCC	pT2 pN0 pMx
8	Oropharynx	6	Primary staging	SCC	pT4b pN2b pMx
9	Buccal mucosa	2	Primary staging	SCC	pT1 pN2b pMx
10	Hypopharynx	6	Primary staging	SCC	pT1 pN1 pMx
11	Tongue	14	Recurrent tumour	SCC	rpT2 pNx pMx
12	Parotid Gland	1	Recurrent tumour	Adenoid cystic Carcinoma	rpT1 pNx pMx
13	Floor of the mouth	1	Primary staging	SCC	pT1 pN0 pMx
14	Tongue	0	Primary staging	SCC	pT1 pN0 pMx
15	Oropharynx	1	Primary staging	SCC	pT1 pN2a pMx
16	Oropharynx	0	Primary staging	SCC	pT3 pN2b pMx
17	Tongue	0	Primary staging	SCC	pT3 pN2b pMx
18	Hypopharynx	0	Primary staging	SCC	pT3 pN2a pMx
19	Tongue	0	Primary staging	SCC	pT1 pN0 pMx
20	Floor of the mouth	0	Primary staging	SCC	pT1 pN0 pMx
21	Floor of the mouth	0	Primary staging	SCC	pT1 pN0 pMx
22	Floor of the mouth	0	Primary staging	SCC	pT2 pN0 pMx
23	Hypopharynx	0	Primary staging	SCC	pT2 pN2b pMx
24	Oropharynx	0	Primary staging	SCC	pT3 pN2b pMx
25	Buccal mucosa	1	Primary staging	Fibroxanthoma	-
26	Floor of the mouth	6	Metachronous tumour	SCC	pT2 pN0 pMx
27	Floor of the mouth	6	Recurrent tumour	SCC	rpT2 pNx pMx
28	CUP	5	Primary staging	SCC	cTx pN2b pMx
29	Tongue	0	Primary staging	SCC	pT2 pN0 pMx
30	CUP	8	Primary staging	SCC	cTx pN2b pMx
31	Tonsil	1	Recurrent tumour	Fibrosis	-
32	Hypopharynx	3	Primary staging	SCC	pT2 pN2b pMx
33	Vallecula	6	Primary staging	SCC	pT1 pN0 pMx

Abbreviations: *M*: man; *W*: woman; *CUP*: cancer of unknown primary; *SCC*: squamous cell carcinoma; *TNM*: tumour classification according to Weber et al.

Surgical treatment included the resection of the tumour and uni- or bilateral neck dissection in 28 cases. In five cases with recurrent tumour disease, only the tumour was resected.

### ^18^F-FDG PET/CT imaging

PET/CT imaging was performed by using a Siemens Biograph Sensation 64 PET/CT scanner (Siemens Healthcare, Erlangen, Germany) equipped with lutetium oxy-orthosilicate (LSO) crystals. The spatial resolution was 4.4 mm at 1 cm and 5.0 mm at 10 cm from the centre of the transverse FOV (field of view) and the sensitivity was 8.1 kcps/MBq at the centre of the FOV. After at least four hours of fasting, the patients received a weight-dependent intravenous injection of 350–500 MBq of 18F-FDG. Blood glucose levels were checked in all patients, before injection, to be below 150 mg/dl. For attenuation-correction purposes and anatomical correlation, a low-dose CT scan (120 keV, 20 mAs, no i.v.-contrast) was acquired in shallow expiration. When clinically indicated, a diagnostic CT (120 kV, 240 mAs, 0.5 s per rotation, 5 mm slice thickness, portal venous phase 80 s after the injection of 80–120 ml i.v. contrast agent [Imeron 300]) was performed. In patients with a diagnostic CT scan, this scan was used for attenuation correction.

PET scan was performed immediately after CT, with a 3-min acquisition per bed position (6–8), by using a three-dimensional acquisition mode.

### MR imaging

Patients underwent MRI in a Magnetom Verio 3 Tesla MRI Scanner (Siemens Medical Solutions, Erlangen, Germany). We obtained unenhanced axial T1-weighted images, enhanced axial T1-weighted and coronal T1-weighted fat-saturated images after gadolinium DTPA injection (Gadolinium-DTPA 0,1 mmol/kg body weight) and T2-weighted fat-suppressed fast-spin-echo (T2-STIR-sequence) images in axial, sagittal and coronal planes in all patients.

### Data processing and retrospective PET-MR image fusion

All attenuation-corrected PET and enhanced T1-weighted MRI series were retrospectively fused by using a commercially available software program (3D Fusion, Siemens Medical Solutions, Erlangen, Germany) on a separate Siemens Workstation (Syngo MMWP, Siemens Medical Solutions, Erlangen, Germany). The software allows retrospective interactive fusion of two different tomographical imaging modalities (datasets) acquired at different time points. After initial loading of the MR dataset in the 3D card, the PET dataset was included using the fusion tool. Hereby, a first initial orientation manual alignment has to be performed in order to roughly match prominent anatomical landmarks (cerebellum, spine tonsils) of the two datasets. After that, the software automatically aligns the two datasets (MR and PET) based on mutual information using the anatomical contours of the loaded datasets (e.g. head, neck etc.). Lastly, fused images were examined and - if necessary - manually fine adjusted for correct alignment by using landmarks such as the cerebellum, the tonsils, the vocal cords and the spine.

### Image analysis

Two observers, namely an experienced head-and-neck imaging radiologist and an experienced nuclear medicine physician, retrospectively reviewed all 33 image sets of the enrolled patients independently. According to a structured protocol, they evaluated the images for whether a tumour was benign or malignant, tumour localisation, bone infiltration (T-staging) and metastases to cervical lymph nodes (N-staging) by using a five-point-scaling system: 1 = most likely benign, 2 = probably benign, 3 = equivocal/indeterminate, 4 = probably malignant and 5 = most likely malignant.

Metastases to locoregional lymph nodes were analysed separately with regard to the ipsi- or contralateral side and the presence of metastases was recorded according to the classification described by Robins et al. [20]. In an attempt to imitate a normal clinical set-up, none of the observers was aware of the histopathological findings or follow-up but was fully informed of the clinical history and actual clinical findings of the patients.

In the analysis of MRI, the primary tumour was assessed for loss of symmetry, tissue shifting, abnormal tissue enhancement, inhomogeneity in tissue architecture, bony infiltration or other infiltration of neighbouring structures and abnormal focal-contrast enhancement (T-staging). A lymph node was considered as positive for metastasis if the short-axis axial diameter was >10 mm. If lymph nodes were smaller than 10 mm but showed signs of central necrosis and rim contrast enhancement, extra-capsular extension or obliteration of surrounding fat planes, they were also counted as positive neck nodes (N-staging).

The analysis of the PET images was performed visually. The corresponding CT images were only used for anatomical orientation and were not analysed separately or as PET/CT. For the visual analysis all PET images were scaled at a SUV (standardised uptake value) of five. All non-physiological focally increased ^18^F-FDG uptake in the head-and-neck region were analysed in detail, according to the five-point-scaling system (see above).

After the separate analysis of PET and MR images, both observers reviewed the imaging data concurrently in a side-by-side fashion on two adjacent screens. Both observers had to agree to a mutual score with regard to T-staging (including bone infiltration) and N-staging according to the five-point-scaling system.

In the third assessment round, both observers analysed the fused PET-MR images and had to again agree to a mutual score regarding the T-staging (including bone infiltration) and N-staging according to the five-point-scaling system.

### Histopathological reference

The type and extent of surgical resection of the tumour and the type of the neck dissection was determined by our head-and-neck surgical team on the basis of staging results and estimated risk of occult metastases [21]. An experienced anatomical pathologist performed the histopathological evaluation of the resected tissue. The assessment was based on the TNM-classification system presented by Weber et al. [22]. All patients were rated as M_x_ as the M-status had no influence on the study design and analysis. Bony infiltration was analysed by direct histological evaluation of the resected bone. If no bone resection was performed and/or the soft tissue specimen next to the tumour was rated as R_0_, the adjacent bony structures next to the tumour were also rated as being not infiltrated.

### Statistical analysis

The results of the histopathological evaluation were correlated with the diagnostic graduation of MRI, PET, side-by-side analysis and retrospective PET-MR image fusion by using receiver operating characteristic (ROC) curve in combination with Area Under the Curve (AUC). For this analysis the scores of the five-point-scaling system were binary-coded (1–3 were rated as benign and 4–5 were rated as malignant). The binary score of the different imaging techniques and the histopathological analysis of specimen were used as variables.

The Youden-Index was used to determine the cut-off point at which both tumor and lymph node involvement scores had the highest correlation with the histopathological specimen.

Correct positive, false positive, correct negative and false negative rates were calculated and the sensitivity and specificity were determined for T-staging and N-staging. With regard to N-staging, differentiation between positive (N_+_) and negative (N_0_) cervical lymph nodes was performed at the patient level. Because of the low patient count, we could not differentiate between primary or recurrent tumour diseases or between CUP syndromes.

The data was analyzed with the "Statistical Package for the Social Sciences" (SPSS for Windows, release 21.0.0. 2013, SPSS Inc). The figures were generated with SPSS.

## Results

### Technique of image fusion

The semi-automatically image registration with manually adjustment for correct alignment took about 10–20 minutes per case before the analysis started. PET-MRI fusions could be performed in all cases with no obvious limitations, even though fine manual readjustments had to be done in some cases after automatic fusion was completed.

### Results of T-staging

Thirty-one (n = 31) suspected tumour lesions were considered for analysis, since, in one patient of the four CUP syndrome cases, a primary tumour had been detected in the tonsil (case 2). In case 26, an additional metachronous tumour became evident in the analysis and was also accounted for the following analysis. Malignant tumours were detected in 28 tissue sections and three were benign in nature.

The overall sensitivity and specificity of T-staging for MRI, PET, side-by-side analysis and PET-MRI fusion are presented in Table  2.

**Table 2:** **Diagnostic results of tumour staging (T-staging) in thirty-one patients**

Imaging modality	Sensitivity	Specificity	Positive predictive value	Negative predictive value
**MRI**	79% (22/28)	66% (2/3)	96% (22/23)	25% (2/8)
**PET**	82% (23/28)	100% (3/3)	100% (23/23)	38% (3/8)
**side-by-side**	86% (24/28)	100% (3/3)	100% (24/24)	43% (3/7)
**PET-MRI Fusion**	89% (25/28)	100% (3/3)	100% (25/25)	50% (3/6)

Out of the seven falsely rated MRI analyses, one benign lesion was falsely rated as malignant and six malignant lesions were falsely rated as benign. Therefore, MRI alone was associated with a positive predictive value of 96% and a negative predictive value of 25%.

In the analysis of PET alone, five actually malignant lesions were falsely rated as benign. Out of these, three lesions had not been detected and two moderately increased up-takes were rated as nonspecific activity. Therefore, PET alone was associated with a positive predictive value of 100% and a negative predictive value of 38%.

In the simultaneous analysis of MRI and PET in a side-by-side fashion, two additional malignant lesions than in MRI alone were detected and one suspicion of malignancy was rejected. Side-by-side analysis was associated with a positive predictive value of 100% and a negative predictive value of 43%.

The analysis of retrospectively fused PET-MR images detected one additional malignancy in a case after tongue reconstruction. Because of the altered anatomical structures, MRI alone was rated as "indeterminate", whereas in the side-by-side analysis, it was rated as probably benign, with only the evaluation of the fused images confirming the malignancy of the suspicious lesion. Retrospective PET-MRI fusion was associated with a positive predictive value of 100% and a negative predictive value of 50%.

However, three malignant tumours remained undetected in all evaluation rounds. One small tumour of the oropharynx (pT1) and one tumour of the buccal mucosa (pT1) were not seen at all. One carcinoma in situ of the left tonsil was rated as 2 = probably benign in MRI and as 1 = most likely benign in PET/CT and in side-by-side analysis and image fusion. Biopsy by panendoscopy revealed these three tumours histologically.

**Figure 1:**
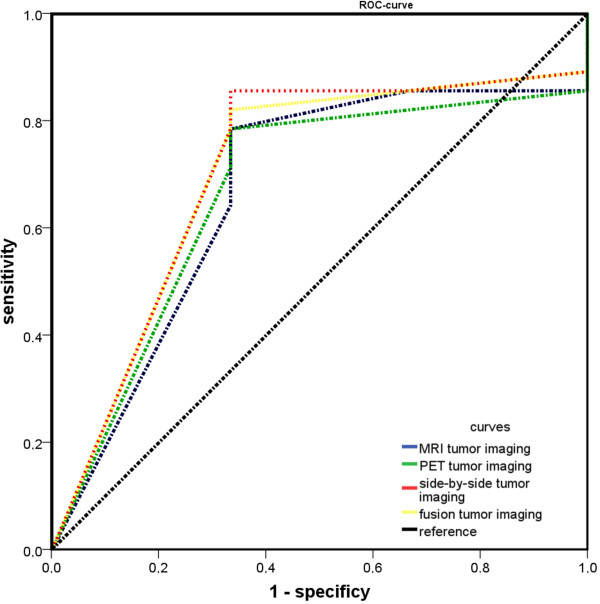
**ROC-curves to compare the accuracy of the different techniques for detection of malignant lesions.**

As a summary statistic the ROC-curve is illustrated in Figure 1. The cut-off point for the different methods investigating tumor evidence was at a score of 3.5 in all methods described. Retrospective image fusion technique (AUC: 0.708, Youden-Index: 0.524) was the most reliable technique, followed by side-by-side analysis (AUC: 0.702, Youden-Index: 0.488) and both the MRI and PET imaging (AUC: 0.667, Youden-Index: 0.453). There were no significant differences in tumor detection between the investigated methods (p > 0.05).

The analysis of mandibular bone infiltration was impaired in single case because of susceptibility artefacts in MRI (n = 2) or because of motion artefacts (n = 1). In PET/CT imaging, the analysis of potentially involved bone was complicated by beam-hardening artefacts in four cases (n = 4), caused by metallic implants. In two out of 28 malignant tumour resections, bony infiltration was established in histological work-up. MRI and PET analysis alone were each able to detect these bone infiltrations but were also associated with one false positive diagnosis each in other cases. Finally, the PET-MRI fusion was able to exclude bone infiltration by matching the suspicious tracer uptake of the PET-scan to the soft tissue tumour site next to the bone and not to the probably periodontal inflamed mandible. In the side-by-side analysis, this case was also rated as "probably infiltrated" because of insufficient virtual matching of the two modalities.

### Results of N-staging

In total, twenty-seven cases (27/33) were analysed for lymph node metastases to the neck (N-staging). No neck dissection had been performed in five cases of recurrent tumour disease and in one case of fibroxanthoma. Histopathological specimens showed metastases to the cervical lymph nodes (N_+_) in 16 patients, whereas eleven cases were free of metastases to cervical lymph nodes (N_0_).

The overall sensitivity and specificity of N-staging on a "per patient" basis for MRI, PET, side-by-side analysis and PET-MRI fusion is represented in Table 3.

**Table 3:** **Diagnostic results of nodal staging of the neck (N-staging) in twenty-seven patients on a "per patient" basis (positive vs. negative neck; N + vs. N0)**

Imaging modality	Sensitivity	Specificity	Positive predictive value	Negative predictive value
**MRI**	94% (15/16)	64% (7/11)	79% (15/19)	88% (7/8)
**PET**	94% (15/16)	91% (10/11)	94% (15/16)	91% (10/11)
**Side by Side**	94% (15/16)	82% (9/11)	88% (15/17)	90% (9/10)
**Fusion**	94% (15/16)	82% (9/11)	88% (15/17)	90% (9/10)

In the analysis of MRI or PET data alone, 15 out of 16 cases were correctly diagnosed with metastatic spread to the cervical lymph nodes. MRI analysis alone detected seven N_0_ necks (7/11) correctly; PET detected ten N_0_ necks (10/11) correctly. Therefore, MRI and PET analysis alone were associated with a positive predictive value of 79% and 94% and a negative predictive value of 88% and 91%, respectively.

In side-by-side analysis of MRI and PET data, two more N_0_ necks were detected than in MRI alone. In the first case, a missing increased up-take of ^18^F-FDG in MRI-diagnosed enlarged lymph nodes led to the correct diagnosis. In another case, an "unsure increased up-take" of ^18^F-FDG led to the wrong PET-diagnosis but could be overruled by a clear MRI diagnosis of non-pathological lymph nodes. The side-by-side analysis detected nine N_0_ necks (9/11) correctly and was associated with a positive and negative predictive value of 88% and 90%.

Retrospective PET-MRI fusion achieved the same results as the side-by-side analysis.

On an N-staging basis, MRI, PET, side-by-side analysis and retrospective image fusion were associated with the displayed correct diagnosis/over-staging/under-staging rates in Table  4. All pathological malign lymph nodes that were not detected by imaging had a maximal diameter smaller than 10 mm.

**Figure 2:**
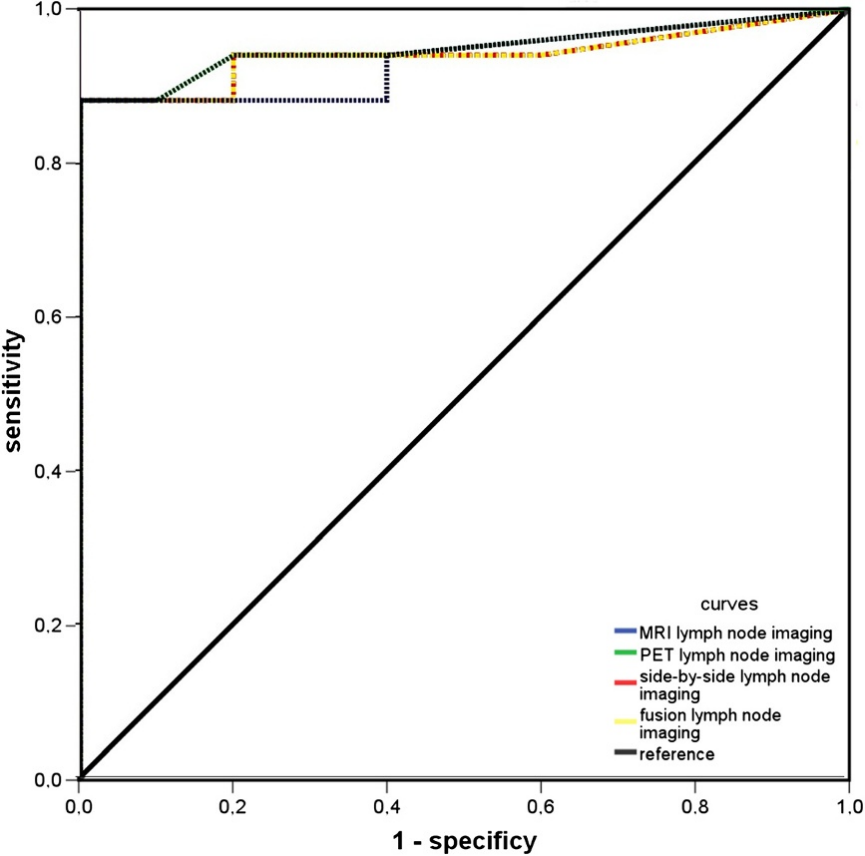
**ROC-curves to compare the accuracy of the different techniques for detection of cervical lymph node metastases.**

The ROC-curve is illustrating the performance of all imaging modalities in detection of lymph node metastases to the neck as a summary statistic (Figure 2). The cut-off point for the different methods investigating lymph node evidence was at a score of 4.5 in all methods described. The most reliable technique was PET imaging (Youden-Index: 0.882, AUC: 0.95), directly followed by side-by-side analysis and retrospective image fusion technique (Youden-Index each: 0.882, AUC: 0.941), which however, were not significantly better then the MRI (Youden-Index each: 0.882, AUC 0.935; p > 0.05).

**Table 4:** **Diagnostic results of nodal staging of the neck (N-staging) in twenty-seven patients by using MRI, PET/CT, side-by-side analysis and retrospective PET-MRI fusion as clinical N-staging with imaging (cN) and correlation to the histopathological results (pN)**

MRI						PET/CT						Side-by-side/PET-MRI Fusion					
	cN0	cN1	cN2a	cN2b	cN2c		cN0	cN1	cN2a	cN2b	cN2c		cN0	cN1	cN2a	cN2b	cN2c
pN0	7	1	0	1	2	pN0	10	1	0	0	0	pN0	9	2	0	0	0
pN1	0	2	0	0	0	pN1	0	2	0	0	0	pN1	0	2	0	0	0
pN2a	0	0	2	0	0	pN2a	0	0	2	0	0	pN2a	0	0	2	0	0
pN2b	1	1	0	7	1	pN2b	1	2	0	6	1	pN2b	1	1	0	7	1
pN2c	0	1	0	0	1	pN2c	0	0	0	0	2	pN2c	0	0	0	0	2
Correct staging: 70.4%						Correct staging: 81.5%						Correct staging: 81.5%					
Over-staging: 18.5%						Over-staging: 7.4%						Over-staging: 11.1%					
Under-staging: 11.1%						Under-staging: 11.1%						Under-staging: 7.4%

## Discussion

In order to determine the clinical benefit of PET-MRI fusion in HNC, we have analysed the value of retrospective PET-MRI fusion and compared the results with side-by-side analysis and PET or MRI alone. Although in our small patient population no statistically significant differences in the performance of each technique could be determined for local tumour assessment or lymph node staging, there was a trend towards a better performance of multimodal imaging (side by side analysis or retrospective image fusion) especially in the diagnosis of recurrent HNC and inconclusive findings in the single modality. Based on these data, this finding now has to be confirmed in larger prospective studies.

To our knowledge, no previous study has compared the value between retrospective image fusion and side-by-side analysis up to now. A diagnostic benefit has to be proven for multimodal imaging, since morphological imaging by using MRI or CT alone has nowadays reached a high accuracy in patient follow-up and even allows the detailed analysis of structures such as mucosal invasion [3, 23]. Accurate assessment of the local tumour extent is important for the surgeon, as the imaging results will determine the extent of tumour resection, the type of reconstruction and the adjuvant therapy.

**Figure 3:**
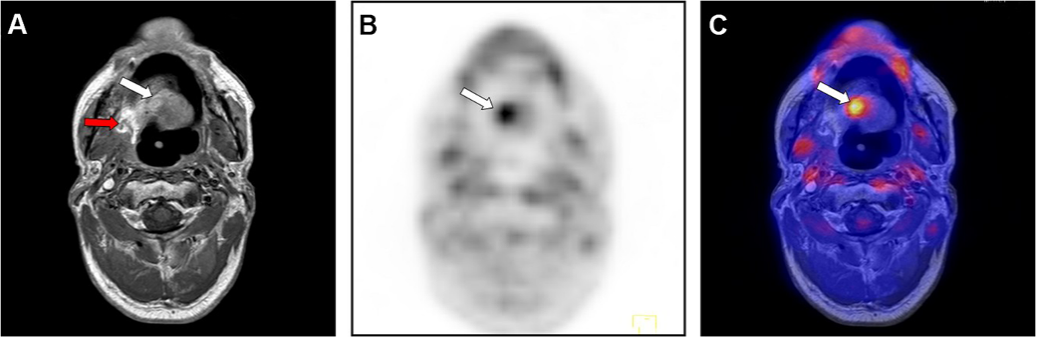
**Beneficial use of retrospective image fusion in a case of recurrent tumour disease.** The presented case (no. 27, rpT2 pNx pMx) has suspected recurrent disease after resection of a squamous cell carcinoma of the floor of the mouth and tongue and reconstruction with a microvascular radial forearm flap and adjuvant radiation. **A**: The tissue by MRI alone was rated as probably malign, attributable to the abnormal contrast enhancement in the right posterior site of the floor of the mouth (red arrow). The region near the midline of the residual tongue (white arrow) was interpreted as an anatomical alteration after surgery and irradiation. **B**: PET alone showed a moderate increase of tracer up-take near the midline and both PET alone and the side-by-side analysis were rated as "probably benign". **C**: Retrospective image fusion provided the correct diagnosis of recurrent tumour disease through the correct alignment of morphological and functional imaging data; however, the disease was present not in the dorso-lateral region (histology: scar fibrosis) but near the midline in the residual tongue (histology: SCC recurrent disease).

In several studies, the beneficial use of PET data in primary T-staging has been reported [24, 25] and has led to the assumption that PET and MRI analysis further complement each other [6, 15]. In our study, one false negative and one false positive rating in MRI could be changed to the correct diagnosis through side-by-side analysis and elevated the sensitivity/specificity to 85%/100%. Retrospective image fusion had the highest sensitivity/specificity rates in T-staging of 89%/100%. It was shown to be of evident value in a suspected recurrent tumour (Figure  3). As is well-known, morphological imaging alone (MRI or CT) in previously operated regions is challenging due to sometimes difficult differentiation between non-neoplastic and neoplastic change. The reasons are scar tissue, loss of symmetry, side shift and unspecific contrast enhancement [5]. PET has gained wide acceptance in staging of suspected recurrent tumour disease, because of its high negative predictive values of up to 95%. On the other hand, it is also associated with false positive findings shortly after operation and should therefore be combined with CT or MRI and should be performed at a time interval of 10–12 weeks after surgery [26–28].

In our analysis, retrospective PET-MRI fusion in initial tumour staging of primary lesions was of minor advantage compared with a single modality or to the side-by-side analysis with regard to T-staging. No significant benefit of retrospective image fusion was seen compared with side-by-side analysis. However, multimodal imaging (fusion and side-by-side analysis) had a tendency for higher sensitivity compared to MRI or PET alone, although the difference was not significant.

Another important aspect of HNC staging is the recognition of tumour invasion of neighbouring structures, such as maxilla or mandible. As described earlier, additional PET data might be beneficial in the detection of bone infiltration compared with MR images alone [29]. In one case of our present study, retrospective PET-MRI fusion could also reduce false positive diagnoses for bone infiltration and correspondingly could reduce surgical over-treatment.

Staging for metastases to cervical lymph nodes (N-staging) plays an important role and correlates directly with the survival of the patient [30, 31]. Correct diagnoses are still associated with difficulties, especially when only morphological imaging technique is performed [32]. The reported sensitivity/specificity ranges between 78-88%/75-86% for MRI alone [4, 33]. The differentiation between benign and malignant lymph node enlargement in morphological imaging alone (CT or MRI) mainly relies on size-based evaluation. However, the sizes of lymph nodes, whether benign or malignant, vary and previously studies have shown that up to 21% of nodes smaller than 10 mm can be malignant and up to 40% of nodes larger than 10 mm can be benign [32, 34]. Our high sensitivity for MRI (94%) can be explained by only differentiating between N_0_ or N_+_ staging and no node-by-node comparison. PET/CT is able to raise the specificity up to 97% but is still accompanied with a low sensitivity of 50-84% [33, 34]. In our results, PET analysis alone achieved the highest sensitivity and specificity rates (94% and 91%) and was higher than that reported in the literature. Our small patient population might well be the reason for these findings.

On a "N-staging per patient basis", MRI only achieved correct staging in 70.4%, whereas PET alone, side-by-side analysis and PET-MRI fusion achieved correct staging in 81.5% (Table  4). Furthermore, side-by-side analysis and retrospective image fusion achieved the lowest under-staging rates compared with PET and MRI alone (7.4% vs. 11.1%). On the other hand, MRI had the highest rates of over-staging (18.5%) compared with multimodal imaging (11.1%). Both retrospective image fusion and side-by-side analysis corrected two initial false positive ratings to N_0_ staging. PET analysis alone was associated with the lowest rate of over-staging (7.4%). As shown in our results, even with retrospective PET-MRI fusion or side-by-side analysis, malignant neck lymph nodes smaller than 10 mm remained undetected and were associated with a lower specificity than by PET analysis alone (82% vs. 91%), as caused by the false positive influence of the MRI. Ng et al. also reported a superiority of PET analysis for nodal staging compared with MRI alone. By analysing the nodal status in a side-by-side fashion, they showed a trend of increased diagnostic accuracy over the single modalities [35].

Our study has some limitations. The main limitations are its retrospective design, the inhomogeneous patient population including patients with primary and recurrent disease and the relatively small number of patients included. However, these limitations do not decisively influence the results of our study. As the observers were not aware of the histopathology report, the retrospective nature of the study should not have influenced the diagnostic performance of image fusion. Furthermore, the aim of our study was to evaluate performance of image fusion of PET and MRI in the head and neck region. We therefore chose to include patients with diseases in this region, independently of the primary or the recurrent disease. Despite the relatively small number of patients included in the study, our results confirmed the known superiority of combining morphological and functional imaging in diagnostic accuracy. However there were no beneficial gains for the image fusion in this aspect compared to side-by-side analysis.

In summary, we found positive trends of multimodal imaging in T- and N-staging of HNC cancer. Nevertheless, the proof of cost-effectiveness of an initial multimodal imaging (PET/CT or PET/MRI) for primary staging of HNC and also any evident and significant advantages in diagnoses of bone infiltration are still pending and must be analysed systematically. Another question to be answered is, whether it is contemporary to perform time consuming and in some cases difficult retrospective image fusion or side-by-side analysis when it is possible to use fully integrated PET/MRI scanners in primary staging of HNC.

## Conclusion

Our study has shown the beneficial use of multimodal imaging by using retrospective PET-MRI fusion in selected HNC cases only. Compared with morphological MRI alone, we have seen advantages in single cases of recurrent diseases and in the ambiguous diagnosis of suspected lymphatic spread to the neck. However, the complex and time-consuming nature of retrospective image fusion hardly justifies the routine use in light of the only slight advantages compared to side-by-side analysis. However this technical limitation can be overcome by fully integrated PET/MRI scanners, which have recently become available. Their clinical value for HNC has not as yet been fully defined [14, 36, 37] and must be analysed systematically in future studies.
